# Sodium Butyrate Attenuates Taurocholate-Induced Acute Pancreatitis by Maintaining Colonic Barrier and Regulating Gut Microorganisms in Mice

**DOI:** 10.3389/fphys.2022.813735

**Published:** 2022-03-17

**Authors:** Yangyang Xiong, Li Ji, Yi Zhao, Ailing Liu, Dong Wu, Jiaming Qian

**Affiliations:** State Key Laboratory of Complex Severe and Rare Diseases, Department of Gastroenterology, Peking Union Medical College Hospital, Peking Union Medical College, Chinese Academy of Medical Sciences, Beijing, China

**Keywords:** acute pancreatitis, sodium butyrate, pancreas, colon, neutrophils, macrophages, gut microorganisms

## Abstract

**Background:**

Acute pancreatitis (AP) damages the intestinal barrier, which aggravates AP. Butyrate exhibits anti-inflammatory effects in AP, but it is unknown if such a protective effect is associated with the regulation of gut microorganisms. We aim to investigate the effects of sodium butyrate (SB) on pancreatic inflammation, colonic barrier, and gut microorganisms.

**Methods:**

C57BL/6 mice were divided into groups of sham operation (Sham), AP, 200 mg/kg SB intervention (SB-200), and 500 mg/kg SB intervention group (SB-500). Samples were harvested 24 h after the model was established. The gut microbiota was analyzed using 16S rRNA gene sequencing.

**Results:**

Pancreatic infiltration of neutrophils, macrophages, and M2-type macrophages was significantly reduced in the SB-500 intervention group. Supplementation of SB-500 improved colon mucosal histology and the expression of ZO-1 and occluding. The relative abundance of *Alloprevotella* and *Muribaculaceae* was increased and that of *Akkermansia* was decreased in the SB-500 group compared with the AP group. *Ruminococcaceae* was the most significantly increased species and *Prevotellaceae* was the most significantly decreased species in the SB-500 group compared with the AP group.

**Conclusion:**

High dose of SB inhibits pancreatic inflammation probably by maintaining the intestinal barrier and regulating gut microbiota in mice with AP.

## Introduction

Acute pancreatitis (AP), an inflammatory condition arising from the pancreas, might lead to severe systemic inflammatory response and multiple organ dysfunction, with a mortality rate up to 50% in severe cases (Pandol et al., 2007; Jha et al., 2009). The pathology of AP is characterized by stromal edema, acinar cell necrosis, and inflammatory infiltration of neutrophils and macrophages (Pandol et al., 2007). In addition, during the early stage of AP, injured acinar cells produce inflammatory factors, such as tumor necrosis factor (TNF)-α, interleukin (IL)-1β, and IL-6 to initiate inflammatory responses (Rodriguez-Nicolas et al., 2018). Some risk factors have been associated with AP, such as gallstones, alcohol abuse, hypertriglyceridemia, hypercalcemia, drugs, and genetic factors (Whitcomb, 2006; Jha et al., 2009; Zheng et al., 2015; Hines and Pandol, 2019). Several methods for the early management of AP have emerged, such as fluid resuscitation (Bakker et al., 2014). However, the current management of AP remains largely supportive and no medication has been proved effective despite dozens of clinical trials (Crockett et al., 2018).

Recent studies have indicated that crosstalk between the pancreas and gut microbiota was associated with the development of pancreatic diseases (Thomas and Jobin, 2020). For example, Zhang et al. (2018) found that gut microbiota differed between patients with AP and healthy individuals. Another study reported that early dysbiosis of the gut microbiota was associated with the occurrence of severe AP (Li et al., 2020). In addition to intestinal microorganisms, some metabolites, such as short-chain fatty acids (SCFAs), also affect the progression of AP. Butyrate is a SCFA produced from the fermentation of dietary fibers by intestinal microbiota (Koh et al., 2016). Accumulating evidence has demonstrated that butyrate had notable effects on gut health by suppressing intestinal inflammation and maintaining intestinal homeostasis (Singh et al., 2014). It was also reported that the intestinal butyric acid-producing flora in patients with AP was significantly reduced, such as *Bacteroides*, *Alloprevotella*, *Blautia*, and *Gemella* (Zhu et al., 2019). Yu et al. (2020) found that butyric acid-producing flora *Eubacterium hallii* was markedly decreased in patients with severe AP compared with that in patients with mild AP and in healthy individuals. It was also noted that sodium butyrate (SB) alleviated pancreatic damage, inflammation, and fibrosis in pancreatitis (Hartman et al., 2015; Kanika et al., 2015). However, the underlying mechanism of SB on AP remains to be elucidated. This study aims to identify the effect of SB on pancreatic inflammation, the colonic barrier, and gut microorganism in an AP mouse model.

## Materials and Methods

### Mice

A total of 32 male C57BL/6 mice (aged 8–10 weeks and weighing 22–25 g) were purchased from Beijing Veitong Lihua Experimental Animal Technology Co., Ltd., Beijing, China. All the mice were fed in the Animal Experimental Center of Peking Union Medical College Hospital, Chinese Academy of Medical Sciences under specific pathogen-free conditions with a 12 h light/dark cycle. The Animal Welfare Ethics Committee of Peking Union Medical College Hospital approved this study (approval no. XHDW-2019-00).

### Experimental Design and Animal Treatments

After 1 week of adaptive feeding, the mice were randomly divided into the following 4 groups (8 mice per group) as follows: phosphate buffer saline (PBS)-treated control group (Sham), sodium taurocholate-induced AP group (AP), SB-treated groups at a dose of 200 mg⋅kg^–1^ (SB-200) and 500 mg⋅kg^–1^ (SB-500) (body weight basis; SB + AP). SB (Sigma-Aldrich, Saint Louis, MO, United States) was administrated intragastrically for 7 days prior to sodium taurocholate injection. For Sham and AP groups, mice were given equivolumetric PBS for 7 days without/with the sodium taurocholate challenge, respectively. The mouse model of AP was established using a retrograde injection of 5% sodium taurocholate into the pancreatic duct (Perides et al., 2010), which is relevant to clinical AP. Twenty-four hours after the sodium taurocholate injection, mice were sacrificed by a lethal dose of pentobarbital sodium (90 mg⋅kg^–1^; Sigma–Aldrich, St Louis, Mosby, MO, United States), and plasma, pancreatic, and colonic tissue samples were harvested for subsequent experiments.

### Detection of Amylase and Lipase in the Serum of Mice

The mice were anesthetized with an intraperitoneal injection of sodium pentobarbital (25–30 mg/kg). The peripheral blood was extracted from the eyeball, the mice were euthanized *via* cervical vertebra dislocation. Peripheral blood specimens were centrifuged at 1,000 g for 5 min at 4°C. The collected serum was stored at –80°C. Chromatography was used to detect the content of Amylase (AMY) and Lipase (LIP) using an Olympus AU5400 automatic biochemical analyzer.

### Hematoxylin and Eosin Staining of Pancreatic and Colon Tissue of Mice

The mice were anesthetized and euthanized *via* cervical dislocation. The pancreatic and colon tissue were fixed overnight at room temperature in 4% formalin solution, paraffin embedded and sectioned into 4 μm within 1 week. The slices were placed in an oven at 60°C for 2 h. After dewaxing, the slices were stained with hematoxylin for 5 min at room temperature and rinsed with running water. After hydrochloric acid ethanol differentiation, eosin staining, dehydration and sealing, the HE staining films were scanned and observed. The images of sections were viewed using CaseViewer 2.4 software (3DHISTEC, Sysmex, Switzerland) and microtissue pathological injury score was calculated. A total of 10 fields (200X magnification) were randomly selected from each slice for scoring and the average value was noted. The scoring criteria used was as previously described (Wildi et al., 2007).

### Detection of Inflammatory Factors and Myeloperoxidase in the Pancreas of Mice

The concentrations of inflammatory cytokines in the pancreatic tissue were determined using ELISA kits (IL-1β, SEKM-0002, Solarbio, China; CXCL1, SEKM-0046, Solarbio, China; TNF-α, SEKM-0034, Solarbio, China; Myeloperoxidase, MPO, SEKM-0118, Solarbio, China) according to the manufacturer’s instructions. Prepared RIPA lysate was added to the samples, thoroughly ground in a homogenizer, centrifuged at 12,500 × *g* for 10 min at 4°C. The supernatant was collected to a new Eppendorf^®^ tube to measure the total protein concentration of the samples using a BCA protein assay Kit (PC0020, Solarbio, China). After the standards were set, the samples were added, followed by the enzymes, and the samples were incubated for 1 h at 37°C. Subsequently, they were washed. The coloration and termination reaction was performed, and then the absorbance was measured at 450 nm within 15 min.

### Detection of the Ratio of Neutrophils and Macrophages in Pancreatic Single-Cell Suspension of the Mice

The pancreatic tissue was digested with digestive fluid to extract the single-cell suspension. The single-cell suspension was centrifuged at 500 *g* for 10 min at 4°C, rinsed once with 1–2 ml precooled FACS solution and centrifuged at 500 *g* for 10 min at 4°C. The single-cell suspension was resuspended in 125 μl FACS solution and stained with trypan blue. Cell viability was observed and counted under a light microscope using a cell counting plate. The resuspended samples were stored on ice for flow cytometry analysis. The neutrophils were labeled with LY6G^+^CD11b^+^ (400532, Biosciences, Franklin Lakes, NJ, United States, 400612, Biosciences, Franklin Lakes, NJ, United States), while the macrophages were labeled with CD11b^+^F4/80^+^ (400612, Biosciences, Franklin Lakes, NJ, United States, 562602, Biosciences, Franklin Lakes, NJ, United States) and the M2-type macrophages were labeled with CD11b^+^ F4/80^+^CD206^+^ (400612 Biosciences, Franklin Lakes, NJ, United States, 562602, Biosciences, Franklin Lakes, NJ, United States, 12-2061-82, Biosciences, Franklin Lakes, NJ, United States). The proportion of the aforementioned cells was detected using flow cytometry and a BD FACSCelesta flow cytometer, and the results were analyzed using FlowJo 10.0 software (Tree Star Incorporation., Ashland, OR, United States).

### Messenger RNA Expression Level Detection of Zona Occludens 1 and Occludin in the Colon Tissue of Mice

Total RNA from colon tissues was extracted using TRIzol reagent (Invitrogen, Carlsbad, CA, United States; Thermo Fisher Scientific Incorporation, Waltham, MA, United States) according to the manufacturer’s instructions. Briefly, colon tissues (100 mg) from mice were collected from each set, taken in 1.5 ml microfuge tube containing 1 ml of TRIzol, and minced finely. The minced tissues were homogenized and incubated for 3–5 min for complete dissociation of nucleoprotein complexes and finally 0.2 ml of chloroform was added per 1 ml of TRIzol. The tubes were inverted several times for 15 s, incubated for 2–3 min at room temperature, and then centrifuged at 12,000 *g* for 15 min at 4°C. The transparent aqueous phase was aspirated out and taken in a new tube and 0.5 ml of 100% isopropanol was added. The tubes were incubated for 1 h at –20°C for precipitation of RNA. The tubes were next centrifuged at 12,000 *g* for 10 min and the supernatant was discarded while the RNA pellet was washed with 0.5 ml of 70% ethanol. This process was repeated once again. The tubes were next centrifuged at 7,500 *g* for 5 min. The supernatant was discarded and the pellet was air-dried to remove the traces of alcohol. The pellet was next dissolved in 50 μl of nuclease free water by incubating at 60°C for 10–15 min and resulting RNA solution was quantified spectrophotometrically. Then, 10 μg of RNA was treated with rDNase (DNA-free kit, Invitrogen, Carlsbad, CA, United States) according to the instructions provided in the kit and finally purified RNA was used for cDNA synthesis. Total RNA (2 μg) was reverse-transcribed using the RevertAid First Strand cDNA synthesis kit (K1622, Invitrogen, Carlsbad, CA, United States; Thermo Fisher Scientific Incorporation, Waltham, MA, United States) according to the manufacturer’s instructions. The cDNA obtained was diluted for PCR. The following primer sequences were used for PCR genotyping: ZO-1, 5′-GCTTTAGCGAACAGAAGGAGC-3′ and 5′-TTCATTTTTCCGAGACTTCACCA-3′, occluding, 5′-TTGAAAGTCCACCTCCTTACAGA-3′ and 5′-CCGGATA AAAAGAGTACGCTGG-3′, GAPDH, 5′-AGGTCGGTGT GAACGGATTTG-3′ and 5′-TGTAGACCATGTAGTTG AGGTCA-3′. The PCR protocol consisted of the following steps using an Applied Biosystems 7500 Fast Real-Time PCR system (Applied Biosystems; Thermo Fisher Scientific Incorporation, Waltham, MA, United States): 95°C for 30 s for initial denaturation, 95°C for 5 s for denaturation, and 40 cycles of extension at 60°C for 30 s. The relative transcription level of Zona Occludens (ZO-1) and occludin was calculated using the 2^–ΔΔ*Ct*^ method.

### Protein Expression Detection of Zona Occludens 1 and Occludin in the Colon Tissue of Mice

Approximately 50 mg of colon tissues were weighed and transferred into a 2 ml tube with 0.5 ml of ice-cold RIPA protein isolation buffer (Beyotime, Shanghai, China). Mixture of colon and RIPA was treated by POLYTRON PT (KINEMATICA, Luzern, Switzerland), a mechanism used for homogenizing tissue in RIPA, for 15 s, incubated on ice for 10 min and then centrifuged at 4°C and 12,000 *g* for 20 min. Then, a new 1.5 ml tube was used to collect the supernatant containing the total protein. The transfer of the supernatant was performed carefully to avoid touching the pellet at the bottom. Then, it was necessary to determine the concentration of the total protein using a BCA Protein Assay kit (Solarbio, Beijing, China). Forty μg of total protein mixed with protein loading buffer was loaded into 10% sodium dodecyl sulfate-polyacrylamide gel electrophoresis (SDS-PAGE) gels to separate the proteins. After separation, SDS-PAGE with blots was stripped and then each blot on the SDS-PAGE was transferred onto nitrocellulose membranes (Pall Gelman Laboratory, Ann Arbor, MI, United States). Immediately, each membrane was blocked at room temperature with Tris-buffered saline with Tween 20 (TBST) containing 7% skim milk powder, or 5% bovine serum albumin (BSA) (Solarbio, Beijing, China) when blocking for phosphorylated protein detection, for 2 h, and then incubated with primary antibodies, including anti-ZO-1 (1:500, ab96587, Rabbit, Abcam, Cambridge, MA, United States), anti-occludin (1:1000, ab216327, Rabbit, Abcam, Cambridge, MA, United States), and anti-GAPDH (1:10000, 60004-1-Ig, Mouse, Proteintech Group, Rosemont, IL, United States). The incubation condition was 4°C overnight. Horseradish peroxidase (HRP)-conjugated secondary antibodies, goat-anti-rabbit (1:10000, 7074s, Goat, CST, Danvers, MA, United States) and goat-anti-mouse (1:10000, 7076s, Horse, CST, Danvers, MA, United States), were incubated at room temperature for 2 h to detect the primary antibodies. After the incubations with the primary and secondary antibodies, the nitrocellulose membranes were washed six times with TBST and each wash time was 10 min. The results were visualized by using High-sig ECL Western Blotting Substrate (Invitrogen, Carlsbad, CA, United States; Thermo Fisher Scientific Incorporation, Waltham, MA, United States). The signals were recorded as optical results by using a ChemiDoc imaging system (Bio-Rad Laboratories Incorporation, Hercules, CA, United States). Bands from three separate western blots were analyzed by Image J software.

### Immunohistochemistry Staining of the Pancreatic and Colon Tissue From the Mice

The immunohistochemical (IHC) analysis was begun using a process that was consistent with other processes for preparing histological sections. The tissue was fixed with paraformaldehyde, dehydrated, embedded in paraffin, and deparaffinized. After deparaffinization, sections for immunohistochemical analysis were treated for antigen retrieval and blocked with 3% H_2_O_2_ for 25 min at room temperature to eliminate endogenous peroxidase activity. The sections were washed with PBS after each step. Then, the sections were blocked with 3% BSA for 30 min at room temperature and incubated with a mouse polyclonal antibody (anti-MPO, 1:800, 22225-1-AP, Rabbit, Proteintech Group, Rosemont, IL, United States; anti-ZO-1, 1:200, ab96587, Rabbit, Abcam, Cambridge, MA, United States; or anti-occludin, 1:100, ab216327, Rabbit, Abcam, Cambridge, MA, United States) overnight at 4°C. After washing with PBS, the sections were incubated with an HRP-conjugated secondary antibody and then washed with PBS. Visualized results of the products were obtained by using a freshly prepared diaminobenzidine (DAB) reaction mixture. Each section was counterstained with hematoxylin for 3 min, dehydrated and covered with a coverslip. The IHC staining images were scanned and observed and viewed using CaseViewer 2.4 software (3DHISTEC, Sysmex, Switzerland). IHC staining result was scored based on the coloration score and the number of positive cells. For the coloration score, 0, 1, 2, and 3 points represented no coloring, light color, moderate coloration, and strong coloring, respectively. For the number of positive cells, 0, 1, 2, and 3 points represented < 25, 25–50, 51–75, and >75%, respectively. The total score was obtained by multiplying the number of positive cells by the degree of comprehensive staining.

### Fecal Sample Collection and Illumina MiSeq Sequencing

To investigate the effect of SB on the intestinal microorganism of the mice with AP, microbial DNA was extracted from the fecal samples of mice in the Sham, AP, and SB-500 groups. Fecal samples were quickly placed into a sterile tube and stored at the –80°C for RNA extraction, PCR, and Illumina MiSeq sequencing.

### Comparative Sample Analysis in the Acute Pancreatitis vs. Sham and SB-500 vs. Acute Pancreatitis Groups

In the comparative sample analysis, beta diversity analysis (sample-level cluster analysis) and sample grouping analysis (partial least squares discriminant analysis; PLS-DA) were included, as described previously (Yu et al., 2020). In the beta diversity analysis, the Bray–Curtis distance algorithm and the average distance method were used for hierarchical clustering analysis. In the sample grouping analysis, PLS-DA can effectively distinguish the observed values between groups and identify the influencing variables that lead to the differences between groups.

### Annotation and Evaluation of Gut Microbiota Species in the Acute Pancreatitis vs. Sham and SB-500 vs. Acute Pancreatitis Groups

To understand the abundance and uniformity of the gut microbiota species, changes in total and core gut microbiota species, and the diversity of the community, rank-abundance and pan/core analysis was performed using R language 3.3.1 software (University of Auckland, New Zealand), as described previously (Yu et al., 2020).

### Analysis of Gut Microbiota Species Composition in Acute Pancreatitis vs. Sham and SB-500 vs. Acute Pancreatitis Groups

In the AP vs. Sham and SB-500 vs. AP groups, the bar chart and Circos diagrams were used to analyze the gut microbiota community composition, and the association between samples and gut microbiota species using R language 3.3.1 software (University of Auckland, New Zealand), as described previously (Yu et al., 2020).

### Analysis of Gut Microbiota Species Difference in the Acute Pancreatitis vs. Sham and SB-500 vs. Acute Pancreatitis Groups

Based on the community abundance data, relevant analysis methods were used to detect the diversity of microbial communities. The non-parametric statistical test was used to screen the different gut microbiota species in the AP vs. Sham and SB-500 vs. AP groups.

### Network Analysis in the Acute Pancreatitis vs. Sham and SB-500 vs. Acute Pancreatitis Groups

To assess whether there were interactions between gut microbiota species in the same phylum between the AP vs. Sham and SB-500 vs. AP groups, gut microbiota species correlation network analysis at the phylum level was performed. The top 50 gut microbiota species in total abundance at the classification level were selected. The correlation was calculated using the Spearman’s correlation method. The correlation coefficient (*r*) > 0.8 was set to establish the correlation network.

### Statistical Analysis

All the data were statistically analyzed using SPSS v22.0 software (IBM Corp., Armonk, NY, United States) and GraphPad Prism v7.0 (GraphPad Software, Inc., La Jolla, CA, United States). The data which were normally distributed were presented as the mean ± standard error of the mean. Pairwise comparison was performed using a one-way ANOVA with a *post hoc* test. The data which were not normally distributed were expressed as the median (and interquartile range). Pair-to-pair comparative analysis was performed using a Mann–Whitney *U* test. Enumeration data were expressed as percentages and compared using a chi-squared test. *P* < 0.05 was considered to indicate a statistically significant difference.

## Results

### Effects of Sodium Butyrate on the Concentration of Amylase and Lipase in the Serum of Mice

**FIGURE 1:**
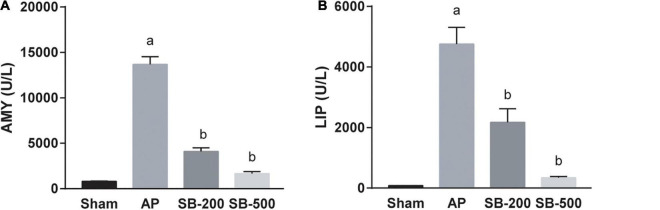
Concentrations of AMY and LIP in the serum of mice in each group. The **(A)** AMY and **(B)** LIP concentration in the serum of mice in each group. *n* = 8. The data was presented as the mean ± standard error of the mean. *^a^p* < 0.05 vs. Sham group. *^b^p* < 0.05 vs. AP group. AMY, amylase; LIP, lipase; Sham, Sham operation group; AP, acute pancreatitis group; SB-200, 200 mg/kg butyric acid intervention group; SB-500, 500 mg/kg butyric acid intervention group.

Figures 1A,B showed that serum concentrations of AMY and LIP in the 200 mg/kg and 500 mg/kg SB intervention groups were both significantly lower compared with those in the AP group in a dose-dependent manner (*p* < 0.05).

### Effects of Sodium Butyrate on the Concentrations of Inflammatory Factors in the Pancreas Tissue of Mice

**FIGURE 2:**
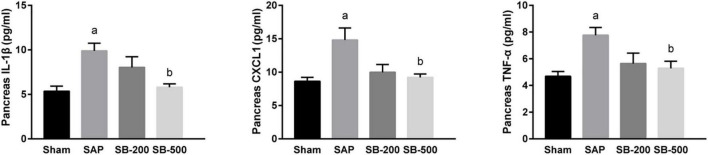
The concentration of IL-1β, CXCL1, and TNF-α in the pancreas of mice in each group. *n* = 8. The data were presented as the mean ± standard error of the mean. *p* < 0.05 vs. Sham group. Sham, Sham operation group; AP, acute pancreatitis group; SB-200, 200 mg/kg butyric acid intervention group; SB-500, 500 mg/kg butyric acid intervention group; TNF, tumor necrosis factor. *^a^p* < 0.05 vs. Sham group. *^b^p* < 0.05 vs. AP group.

Figure 2 showed that pancreatic concentrations of IL-1β, CXCL1, and TNF-α in the AP group were significantly higher compared with those in the sham group (*p* < 0.05). In addition, the concentrations of IL-1β, CXCL1, and TNF-α in the pancreatic tissues from the SB-500 group were significantly decreased compared with those in the AP group (*p* < 0.05).

### Effects of Sodium Butyrate on the Proportion of Neutrophils and Macrophages in Mouse Pancreatic Tissue

**FIGURE 3:**
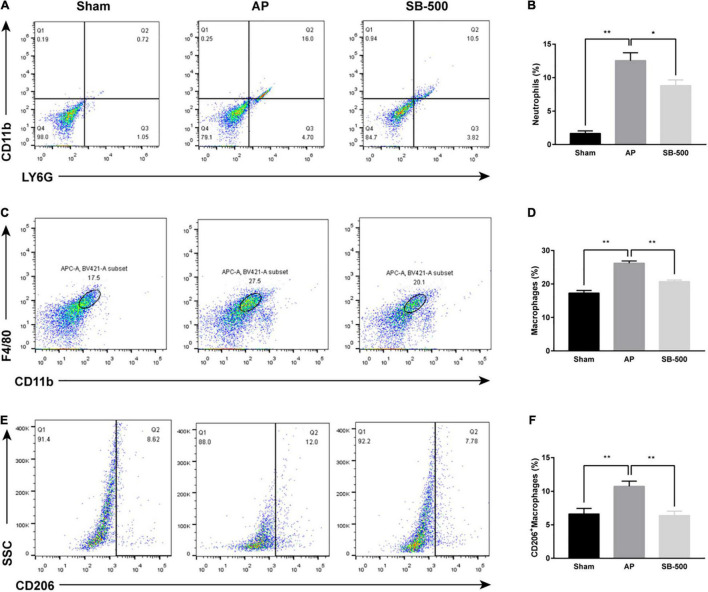
Changes in the proportion of neutrophils, macrophages, and M2 type macrophages in the pancreatic tissue of mice. **(A)** Neutrophils were labeled with LY6G^+^CD11b^+^. **(B)** Changes in the proportion of LY6G^+^CD11b^+^ labeled neutrophils in pancreatic tissue of mice in each group. **(C)** CD11b^+^F4/80^+^ was used to label macrophages. **(D)** Changes in the proportion of CD11b^+^F4/80^+^ labeled macrophages in the pancreatic tissue of mice in each group. **(E)** M2-type macrophages were labeled with CD11b^+^F4/80^+^CD206^+^. **(F)** Changes in the proportion of CD11b^+^F4/80^+^CD206^+^ labeled M2-type macrophages in pancreatic tissue of mice in each group. *n* = 6. The data was presented as the mean ± standard error of the mean. *p < 0.05; **p < 0.01. Sham, Sham operation group; AP, acute pancreatitis group; SB-500, 500 mg/kg butyric acid intervention group.

The proportion of neutrophils, macrophages, and M2-type macrophages in the pancreatic tissue in the AP group was significantly increased than the Sham group (*p* < 0.05), but was significantly reduced in the SB-500 group was significantly decreased (*p* < 0.05) (Figure 3).

### Effects of Sodium Butyrate on Inflammation in the Pancreatic Tissue From Mice

**FIGURE 4:**
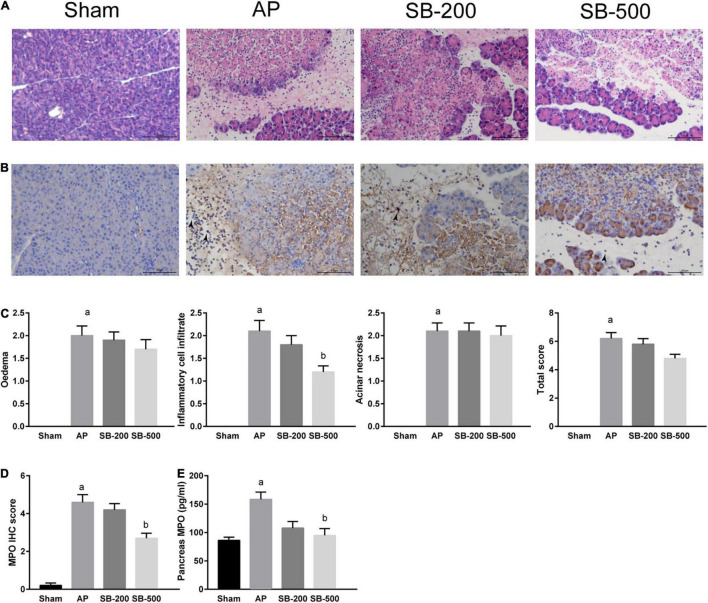
HE and IHC staining, and concentration of MPO in the pancreatic tissue of mice in each group. **(A)** HE staining of the pancreatic tissue from mice in each group (x200). **(B)** IHC staining of MPO in the pancreatic tissue from mice in each group (x200). The black arrow indicates MPO positive cells. **(C)** Pathological score of HE staining of the pancreatic tissue from mice in each group. **(D)** IHC staining score of MPO in the pancreatic tissue in mice in each group. **(E)** Concentration of MPO in the pancreatic tissue from mice in each group. *n* = 8. The data were presented as the mean ± standard error of the mean. ^a^*p* < 0.05 vs. Sham group. *^b^p* < 0.05 vs. AP group. Sham, Sham operation group; AP, acute pancreatitis group; SB-200, 200 mg/kg SB intervention group; SB-500, 500 mg/kg SB intervention group; IHC, immunohistochemical staining; MPO, myeloperoxidase; HE, hematoxylin and eosin.

Figure 4A showed that in the AP group, the normal pancreatic acinar structure disappeared, with diffuse necrosis of the acinar cells, evident interlobular septal edema, and notable inflammatory cell infiltration and cellulose exudation. Compared with that in the AP group, inflammatory cell infiltration, and cellulose exudation were significantly reduced in the SB-500 group, whereas the interlobular septal edema was slightly reduced, but the necrosis of pancreatic cells were not significantly changed (Figures 4A,C). Inflammatory cell infiltration and necrosis of the pancreas in the SB-200 group was not significantly changed compared with that in the AP group (Figures 4A,C). Myeloperoxidase (MPO) IHC staining showed that the number of MPO positive cells in the pancreatic tissue from the SB-500 group was significantly lower compared with that in the AP group. There was no statistical difference in the number of MPO positive cells between the SB-200 group and AP group (Figures 4B,D). The concentration of MPO in the pancreatic tissue from ELISA was consistent with the aforementioned results (Figure 4E).

### Effects of Sodium Butyrate on the Colonic Barrier Function in Mice

**FIGURE 5:**
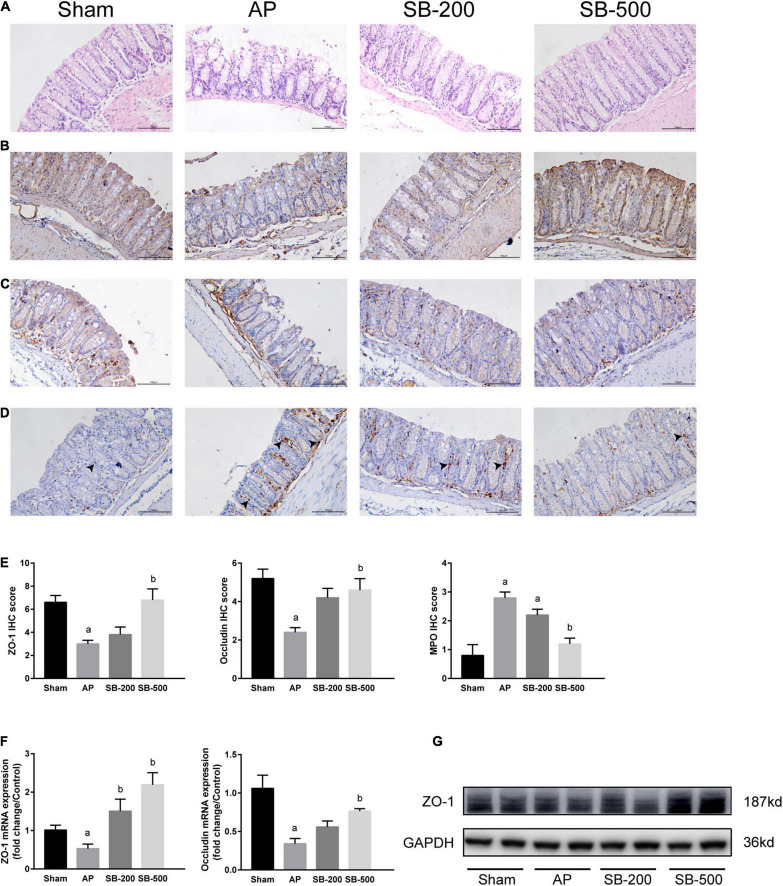
Colonic barrier functions changes in the mice from each group. **(A)** HE staining of colon tissue from mice in each group (×200). **(B)** IHC staining of ZO-1 in colon tissue from mice in each group (×200). **(C)** IHC staining of occludin in the colon tissue from mice in each group (×200). **(D)** IHC staining of MPO in the colon tissue from mice in each group (×200). The black arrow indicates MPO positive cells. **(E)** IHC staining scores of ZO-1, occludin, and MPO in the colon tissue in each group. The **(F)** mRNA and **(G)** protein expression levels of ZO-1 and occludin in the colon tissues from mice in each group. *n* = 8. The data were presented as the mean ± standard error of the mean. ^a^*p* < 0.05 vs. Sham group. *^b^p* < 0.05 vs. AP group. Sham, Sham operation group; AP, acute pancreatitis group; SB-200, 200 mg/kg butyric acid intervention group; SB-500, 500 mg/kg butyric acid intervention group; IHC, immunohistochemical staining; MPO, myeloperoxidase; HE, hematoxylin and eosin; ZO, zona occludins.

As showed in Figure 5, colonic tissue from the mice in the Sham operation group was normal. In the AP group, colonic villi became shorter, the brush margin was destroyed and inflammatory cell infiltration was significantly increased compared with that in the Sham group (Figures 5A,D,E). The villi in the SB-500 group were significantly longer and inflammatory cell infiltration was significantly reduced compared with that in the AP group (Figures 5A,D,E). As showed in Figure 5, the protein expression levels of ZO-1 and occludin in the AP group were significantly decreased compared with those in the Sham group (*p* < 0.05). Compared with those in the AP group, the protein expression levels of ZO-1 and occludin in the SB-500 group were significantly increased (*p* < 0.05) (Figures 5B,C,E,G). Reverse transcription-qPCR results showed that the mRNA expression levels of ZO-1 and occludin in the AP group were significantly decreased compared with those in the Sham group (*p* < 0.05). The mRNA expression levels of ZO-1 and occludin in the colon tissues from the SB-500 group were significantly higher compared with those in the AP group (*p* < 0.05) (Figure 5F).

### Comparative Sample Analysis in the Acute Pancreatitis vs. Sham and SB-500 vs. Acute Pancreatitis Groups

**FIGURE 6:**
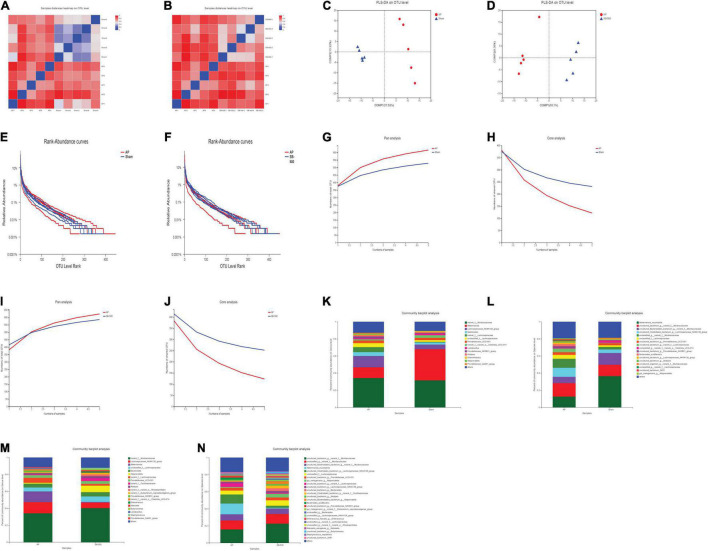
**(A,B)** The heat map of the correlation of samples in the beta diversity analysis in the AP vs. Sham and SB-500 vs. AP groups. **(A)** AP vs. Sham group. **(B)** SB-500 vs. AP group. The distance between samples is represented by a certain color gradient, which is shown on the right. The size of the distance can be seen from the similar color areas. The closer to the red color, the smaller the difference between the two samples. Sham, Sham operation group; AP, acute pancreatitis group; SB-500, 500 mg/kg butyric acid intervention group. **(C,D)** Sample grouping analysis (PLS-DA) in the AP vs. Sham and SB-500 vs. AP groups. **(C)** AP vs. Sham group. **(D)** SB-500 vs. AP group. Sham, Sham operation group; AP, acute pancreatitis group; SB-500, 500 mg/kg butyric acid intervention group; PLS-DA, partial least squares discriminant analysis. **(E,F)** Rank-abundance analysis in the AP vs. Sham and SB-500 vs. AP groups. The *X* and *Y* axes represent the operational taxonomic unit (OTU) level rank and relative abundance, respectively. Sham, Sham operation group; AP, acute pancreatitis group; SB-500, 500 mg/kg butyric acid intervention group. **(G–J)** Pan/Core analysis in the **(G,H)** AP vs. Sham and **(I,J)** SB-500 vs. AP. The *X* and *Y* axes represent the number of observed samples and the number of OTU shared by all samples in a grouping category, respectively. Pan OTU and core OTU represent the union number of common OTU and the intersection number of common OTU, respectively. Sham, Sham operation group; AP, acute pancreatitis group; SB-500, 500 mg/kg butyric acid intervention group. **(K–N)** The community in the bar chart of the **(K,L)** AP vs. Sham and **(M,N)** SB-500 vs. AP groups. The left and right panels represent the genus and species levels, respectively. Sham, Sham operation group; AP, acute pancreatitis group; SB-500, 500 mg/kg butyric acid intervention group.

The beta diversity analysis showed the correlation between the samples in the AP vs. Sham (Figure 6A) and SB-500 vs. AP (Figure 6B) groups, which indicated that heterogeneity between these samples was small. Sample grouping analysis (PLS-DA) showed a clear separation between these groups (Figures 6C,D). This suggested that the structure of gut microbiota was significantly different between these samples.

### Gut Microbiota Species Annotation and Evaluation in the Acute Pancreatitis vs. Sham and SB-500 vs. Acute Pancreatitis Groups

Rank-abundance analysis showed that the gut microbiota species in these samples were abundant and evenly distributed (Figures 6E,F). As the number of samples increased, the total number of gut microbiota species also increased, while the number of core gut microbiota species decreased in these samples in the pan/core analysis (Figures 6G–J).

### Gut Microbiota Species Composition in the Acute Pancreatitis vs. Sham and SB-500 vs. Acute Pancreatitis Groups

**FIGURE 7:**
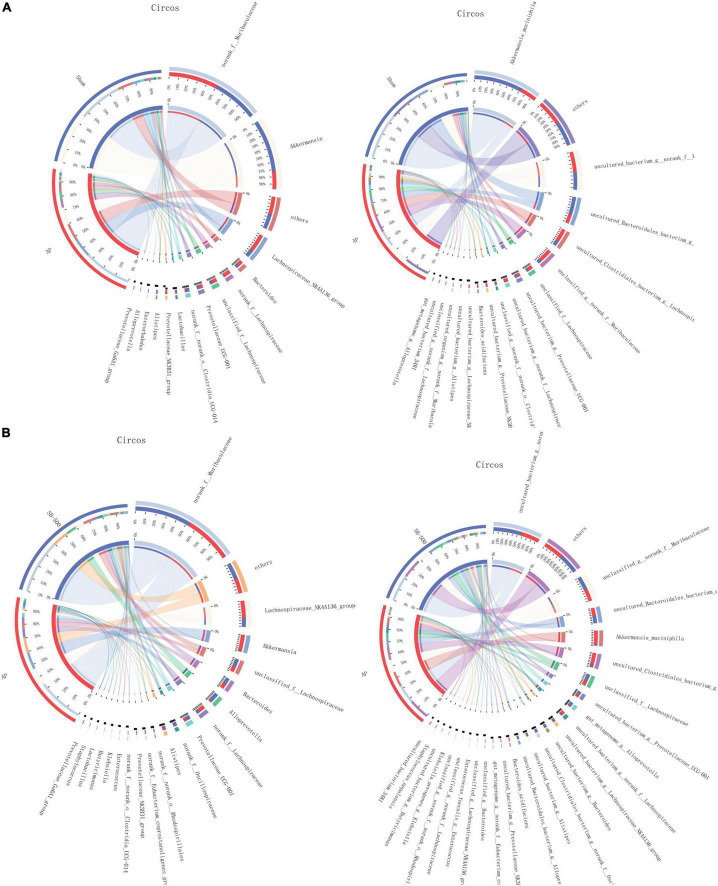
Distribution of the predominant dominant gut microbiota species at the genus and species levels in Circos in the **(A)** AP vs. Sham and **(B)** SB-500 vs. AP, at the (a) genus and (b) species level. Sham, Sham operation group; AP, acute pancreatitis group; SB-500, 500 mg/kg butyric acid intervention group.

The community of gut microbiota in the bar chart showed that the relative abundance of *norank f Muribaculaceae* and *Akkermansia* was increased and decreased, respectively, at both the genus and species levels in the AP group compared with that in the Sham group (Figures 6K,L). The relative abundance of *Alloprevotella* (at the genus level) and *Muribaculaceae* (at the species level) was increased and the relative abundance of *Akkermansia* was decreased at both the genus and species levels in the SB-500 group compared with the AP group (Figures 6M,N). From the Circos diagram, it was found that *Lachnospiraceae* was the dominant gut microbiota species at both the genus and species levels in the AP group compared with that in the Sham group (Figure 7A). *Alloprevotella* was the dominant gut microbiota species at both the genus and species levels in the SB-500 group compared with that in the AP group (Figure 7B).

### Gut Microbiota Species Difference in the Acute Pancreatitis vs. Sham and SB-500 vs. Acute Pancreatitis Groups

**FIGURE 8:**
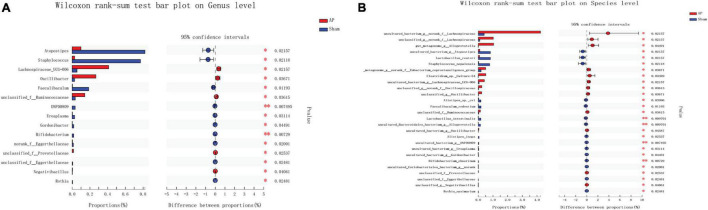
Analysis of gut microbiota species difference at the genus and species levels in the AP vs. Sham group. At the **(A)** genus and **(B)** level. On the left, the *X* and *Y* axes represent the average relative abundance of the gut microbiota species in various groups and the gut microbiota species names at a certain classification level, respectively. On the right, the *X* and *Y* axes represent different gut microbiota species between various groups and *p*-value of significance. *0.01 < *p* < 0.05. ^**^0.01 < *p* < 0.001. Sham, Sham operation group; AP, acute pancreatitis group.

**FIGURE 9:**
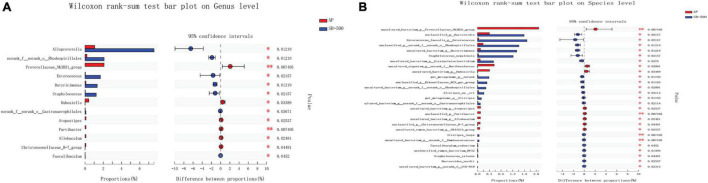
Analysis of gut microbiota species difference at the genus and species levels in the SB-500 vs. AP. At the **(A)** genus and **(B)** species level. On the left, the *X* and *Y* axes represent the average relative abundance of the gut microbiota species in various groups and the gut microbiota species names at a certain classification level, respectively. On the right, the *X* and *Y* axes represent different gut microbiota species between various groups and *p*-value of significance. *0.01 < *p* < 0.05. ^**^0.01 < *p* < 0.001. AP, acute pancreatitis group; SB-500, 500 mg/kg butyric acid intervention group.

*Lachnospiraceae* and *Bifidobacterium* were the most significantly increased (*p* ≤ 0.05) and decreased (*p* ≤ 0.01) species, respectively, at both the genus and species levels in the AP group compared with the Sham group (Figure 8). *Alloprevotella* (at the genus level) (*p* ≤ 0.05) and *Ruminococcaceae* (at the species level) (*p* ≤ 0.01) were the most significantly increased species, while *Prevotellaceae* (*p* ≤ 0.01) was the most significantly decreased species at both the genus and species levels in the SB-500 group compared with the AP group (Figure 9).

### Network Analysis in the Acute Pancreatitis vs. Sham and SB-500 vs. Acute Pancreatitis Groups

**FIGURE 10:**
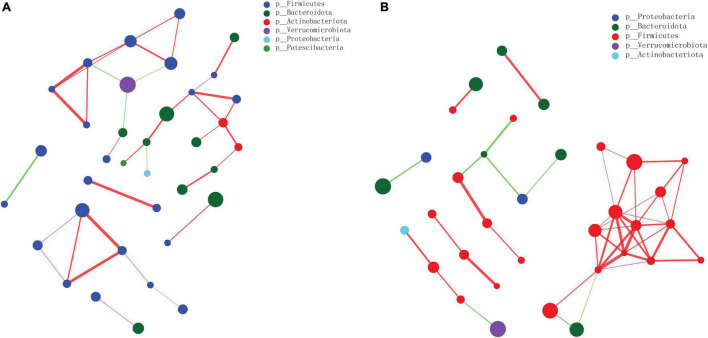
The gut microbiota species correlation network analysis at the phylum level in the **(A)** AP vs. Sham and the **(B)** SB-500 vs. AP groups. The size of nodes indicates the abundance of gut microbiota species; the thickness of the line indicates the value of correlation coefficient; the color of the line indicates the positive correlation, the red and green indicate the positive and negative correlation between gut microbiota species, respectively. SB-500, 500 mg/kg butyric acid intervention group; AP, acute pancreatitis group.

There was a total of 6 and 5 gut microbiota phyla in the gut microbiota species correlation network map in the AP vs. Sham (Figure 10A) and SB-500 vs. AP (Figure 10B), respectively. Notably, there was an interaction between *Bacteroidota*, *Firmicutes*, and *Verrucomicrobiota* in the aforementioned groups.

## Discussion

In this study, we demonstrated that supplementation of high dose SB displayed protective effects against AP in mice by reducing pancreatic inflammation, improving intestinal mucosal histology, and attenuating intestinal permeability. More importantly, alteration of the abundance and species of gut microorganisms by SB intervention may be beneficial for the prognosis of AP.

Neutrophils have been associated with the development of severe AP, and can promote the recruitment of immune cells to the pancreas and propagate tissue inflammation and damage (Yang et al., 2015). Notably, neutrophil depletion significantly reduced tissue damage severity in AP, indicating an important role of neutrophils for tissue infiltration (Abdulla et al., 2011). Macrophages play a key role in numerous inflammatory disorders and are mainly divided into two subpopulations, M1 and M2 macrophages. Changes with M2 polarization enhanced the inflammatory status of macrophages during AP (Bonjoch et al., 2015). In addition, activated macrophages also released proinflammatory cytokines, such as TNF-α, IL-1β, and IL-6, in response to the local damage of the pancreas (Neoptolemos et al., 1998). In this study, we found that SB intervention significantly reduced the proportion of neutrophils, macrophages, and M2-type macrophages in the pancreatic tissue from mice with AP. In addition, supplementation with a large dose of SB inhibited IL-1β, CXCL1, and TNF-α level. These findings suggest that SB played a protective role in the regulation of AP, which is consistent with the aforementioned studies.

Impaired bowel barrier function is common in patients with AP, often leading to infected pancreatic necrosis, sepsis, and organ failure (van Minnen et al., 2007). Intestinal barrier includes mucus barrier, mechanical barrier, biological barrier, immune barrier, and intestinal motility. The mechanical barrier, which is mainly composed of a layer of tightly arranged monolayer epithelial cells, plays an essential role, and has close connections between adjacent cells (such as ZO-1 and occludin) at the top of cells (Huber and Weis, 2001; Suzuki, 2013). Decreased expression of ZO-1 has been found in the ileal mucosa in severe AP (Su et al., 2019). Decreased occludin contributed to impaired intestinal barrier during severe AP (Zhang et al., 2017). In this study, we found that SB intervention upregulated the expression of ZO-1 and occludin in the mice with AP. By promoting the expression of intestinal trilobite factors, SB supplementation helps maintain the integrity of the intestinal mucosal barrier (Jiminez et al., 2017). In the rat model of sepsis, exogenous SB supplementation reduced intestinal permeability, attenuated intestinal injury, and improved prognosis (Fu et al., 2019). Tong et al. (2016) found that SB could improve intestinal barrier function by increasing the expression level of ZO-1 and occludin in the colon tissue from a colitis mouse model. These studies and our results all suggest that exogenous SB supplementation can reduce inflammation and improve intestinal barrier function in AP.

We also investigated the effect of SB on gut microorganism in AP. According to the analysis of gut microbiota species composition, the relative abundance of *Alloprevotella* (at the genus level) and *Muribaculaceae* (at the species level) was increased and the relative abundance of *Akkermansia* was decreased at both the genus and species levels in the SB-500 group compared with the AP group. Furthermore, *Alloprevotella* was the dominant gut microbiota species at both the genus and species levels in the SB-500 group compared with that in the AP group. *Alloprevotella*, gram-negative and SCFA-producing bacteria, was found in isolates from endodontic infections in active dental caries (Schulze-Schweifing et al., 2014). *Alloprevotella* mainly also produces butyric, acetic, and succinic acids (Downes et al., 2013; Cheng et al., 2019), and was involved in stimulating gastrointestinal motility to protect the gut mucosal barrier and insulin resistance (Zhou et al., 2020). In the gut, *Muribaculaceae* (beneficial bacteria) was associated with SCFA and butyrate production with enrichment in *Muribaculaceae* (Meng et al., 2019; Smith et al., 2019; Sibai et al., 2020; Wang et al., 2020). *Muribaculaceae* also plays an important role in the regulation of the community composition and metabolites of microbial flora. *Akkermansia* is a gram-negative bacterium in the human intestinal tract and affected competition among hydrogenotrophic bacteria, which has an impact on production of butyrate (Endesfelder et al., 2016). *Akkermansia* was found to be higher in patients with chronic pancreatitis (Wu et al., 2021). Our findings suggested that SB may promote the production of beneficial bacteria (*Alloprevotella* and *Muribaculaceae*) and reduce the production of harmful bacteria (*Akkermansia*) in AP.

Based on the gut microbiota species difference analysis, it was found that *Bifidobacterium* was the most decreased species at both the genus and species levels in the AP group compared with that in the Sham group. *Bifidobacterium*, a well-known gram-positive probiotic, improved the nutritional status and enhanced the immunity of patients with severe AP (Jin et al., 2018). In addition, it was found that *Ruminococcaceae* (at the species level) was the most significantly increased species and *Prevotellaceae* was the most significantly decreased species at both the genus and species levels in the SB-500 group compared with that in the AP group. *Ruminococcaceae* is a known butyrate producer and influences SCFA levels (Louis et al., 2010). *Ruminococcaceae* was also associated with the release of inflammatory factors from the gut for maintenance of the stable intestinal microecology (Cheng et al., 2017; Ma et al., 2017; Wu et al., 2017). *Prevotellaceae*, a pathogenic bacterium that causes chronic intestinal inflammation, is more abundant in patients with chronic pancreatitis and severe AP (Han et al., 2019; Rehaume et al., 2019). The population of *Prevotellaceae* decreased following administration of SB (Zhang et al., 2016). This indicated that SB may inhibit the inflammatory response by regulating the abundance and content of gut microorganism in AP.

Notably, it was found that there was an interaction between *Bacteroidota*, *Firmicutes*, and *Verrucomicrobiota* in the AP vs. Sham and SB-500 vs. AP groups. *Firmicutes*, the pathogenetic gram-positive bacteria, are mainly butyrate producers and *Firmicutes* has been found in patients with chronic pancreatitis and AP (Hu et al., 2017; Zhang et al., 2018). *Verrucomicrobiota* was the most abundant germ in gut microbiota (Zhou et al., 2020). This indicated that these species could form a specific relationship in the development AP.

There are limitations to this study. First, it is needed to confirm results by using the L-arginine model of AP in the further study. Second, it is required to detect the protein expression of occludin for further study the effects of SB on the colonic barrier function. Third, we did not investigate the detailed protective mechanism of SB on the colon and the pancreas. Fourth, the regulating mechanism upon neutrophil migration by SB remains to be elucidated, and the changes and effects of SB in human AP have not been explored. Fifth, flora transplantation technology is further needed to verify the effects of SB.

Given that no medication has been proved effective in the treatment of AP, translational studies about therapeutic use of butyrate are eagerly needed in this area.

## Conclusion

This study showed that supplementation of SB reduced pancreatic inflammation in AP mice probably by improving intestinal barrier and maintaining gut microbiota homeostasis. These results suggested that SB could be a potential therapeutic agent for AP awaiting further studies.

## Data Availability Statement

The original contributions presented in the study are included in the article/Supplementary Material, further inquiries can be directed to the corresponding author/s.

## Ethics Statement

The animal study was reviewed and approved by the Animal Welfare Ethics Committee of Peking Union Medical College Hospital (approval no. XHDW-2019-00).

## Author Contributions

LJ and YZ analyzed the data. YX and AL interpreted the data. YX was major contributors in writing the manuscript. DW and JQ designed the project. All authors read and approved the final manuscript.

## Conflict of Interest

The authors declare that the research was conducted in the absence of any commercial or financial relationships that could be construed as a potential conflict of interest.

## Publisher’s Note

All claims expressed in this article are solely those of the authors and do not necessarily represent those of their affiliated organizations, or those of the publisher, the editors and the reviewers. Any product that may be evaluated in this article, or claim that may be made by its manufacturer, is not guaranteed or endorsed by the publisher.
