# Supplementary figures and images for: Sodium Butyrate Attenuates Taurocholate-Induced Acute Pancreatitis by Maintaining Colonic Barrier and Regulating Gut Microorganisms in Mice
Source: Front Physiol. 2022 Mar 17;13:813735. doi: 10.3389/fphys.2022.813735 (PMC8969109; doi:10.3389/fphys.2022.813735)

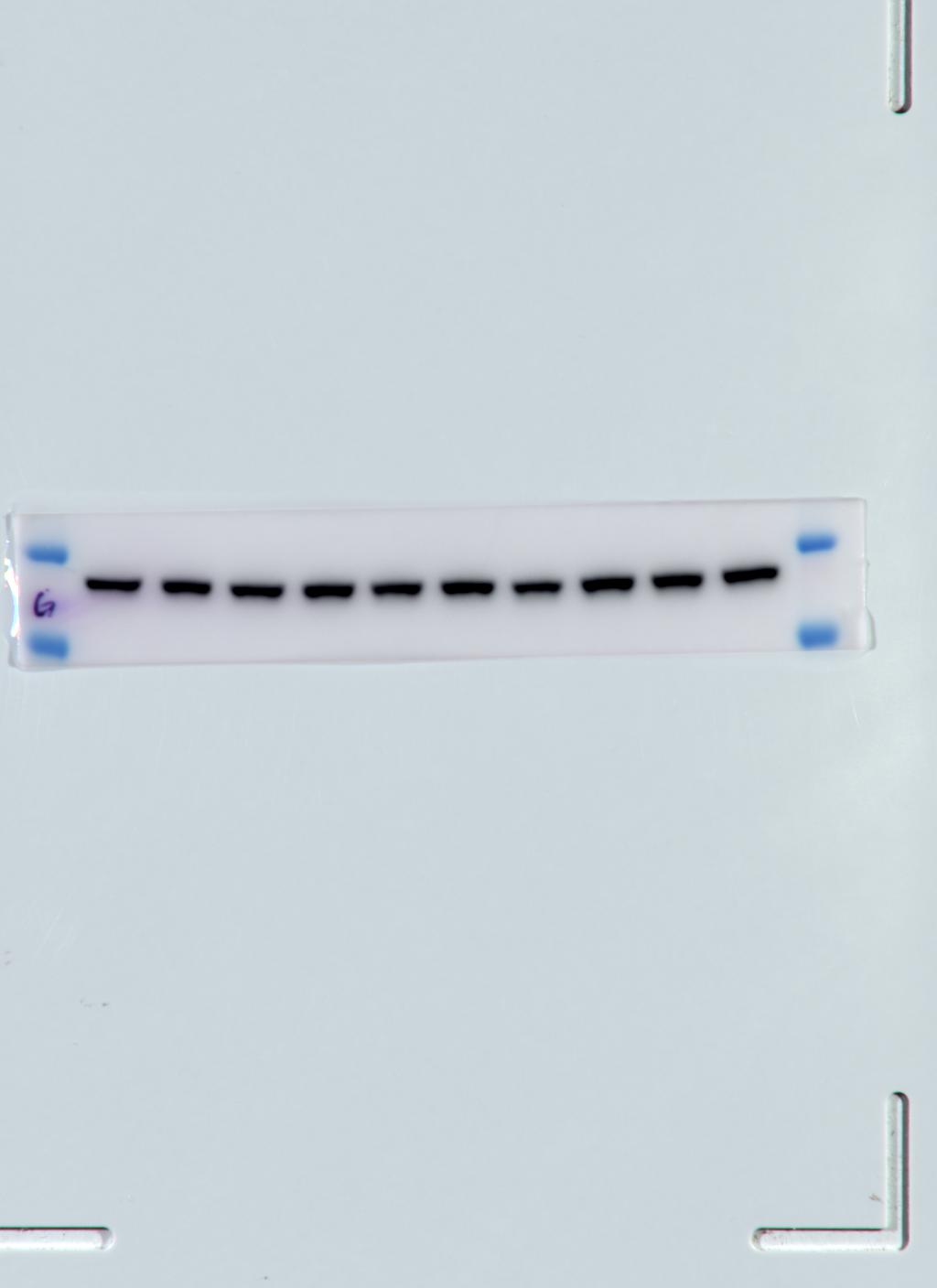

Supplement: Supplementary file 2 [file Data_Sheet_2.ZIP › western blot data/XYY-GAPDH-COLON45 2020.04.16_04.07.49_Ch+Marker.jpg]

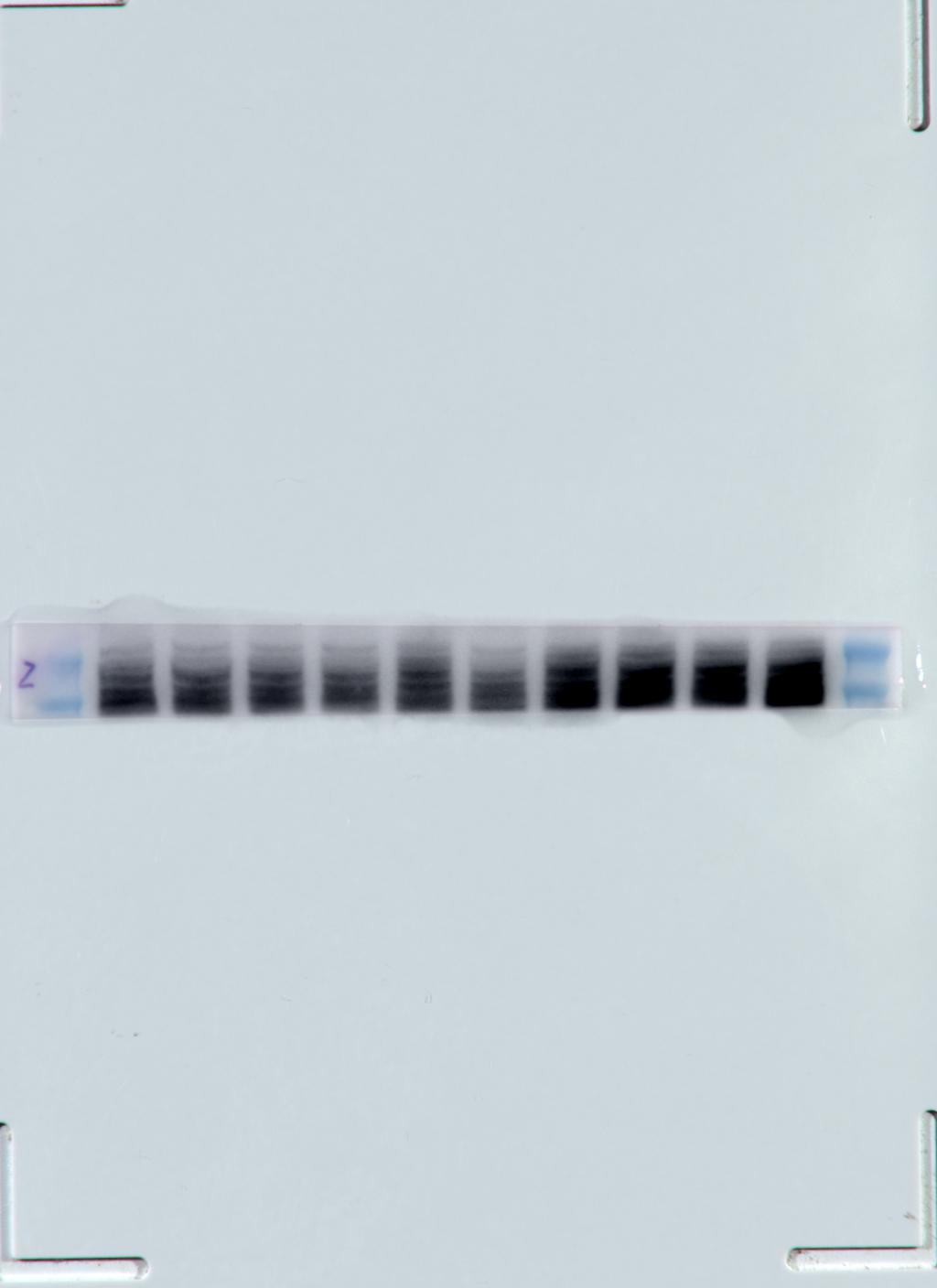

Supplement: Supplementary file 2 [file Data_Sheet_2.ZIP › western blot data/XYY-ZO1-COLON45-0.05S 2020.04.16_05.00.49_Ch+Marker.jpg]
